# Differential regulation of extracellular matrix proteins in three recurrent liver metastases of a single patient with colorectal cancer

**DOI:** 10.1007/s10585-020-10058-8

**Published:** 2020-10-24

**Authors:** Hannah Voß, Marcus Wurlitzer, Daniel J. Smit, Florian Ewald, Malik Alawi, Michael Spohn, Daniela Indenbirken, Maryam Omidi, Kerstin David, Hartmut Juhl, Ronald Simon, Guido Sauter, Lutz Fischer, Jakob R. Izbicki, Mark P. Molloy, Björn Nashan, Hartmut Schlüter, Manfred Jücker

**Affiliations:** 1 Institute of Clinical Chemistry and Laboratory Medicine, University Medical Center Hamburg-Eppendorf, Hamburg, Germany; 2 Institute of Biochemistry and Signal Transduction, University Medical Center Hamburg-Eppendorf, Martinistraße 52, 20246 Hamburg, Germany; 3 Department of General, Visceral and Thoracic Surgery, University Medical Center Hamburg-Eppendorf, Hamburg, Germany; 4 Bioinformatics Core, University Medical Center Hamburg-Eppendorf, Hamburg, Germany; 5 Virus Genomics, Heinrich Pette Institute, Leibniz Institute for Experimental Virology, Hamburg, Germany; 6 Indivumed GmbH, Hamburg, Germany; 7 Institute of Pathology, University Medical Center Hamburg-Eppendorf, Hamburg, Germany; 8 Department of Hepatobiliary and Transplant Surgery, University Medical Center Hamburg-Eppendorf, Hamburg, Germany; 9 Bowel Cancer and Biomarker Laboratory, Faculty of Medicine and Health, The University of Sydney, Sydney, Australia; 10 Present Address: Clinic of Hepato-Pancreatico-Biliary Surgery and Transplantation, First Affiliated Hospital, University of Science and Technology of China, Hefei, People’s Republic of China

**Keywords:** Colorectal cancer, Colorectal liver metastasis, CRC, CRLM, Metachronous liver metastasis, Proteomics, Extracellular matrix, ECM, ECM signatures, Prognostic factor

## Abstract

Colorectal cancer (CRC) patients suffer from the second highest mortality among all cancer entities. In half of all CRC patients, colorectal cancer liver metastases (CRLM) can be observed. Metastatic colorectal cancer is associated with poor overall survival and limited treatment options. Even after successful surgical resection of the primary tumor, metachronous liver metastases occur in one out of eight cases. The only available curative intended treatment is hepatic resection, but metachronous CRLM frequently recur after approximately 1 year. In this study, we performed a proteome analysis of three recurrent liver metastases of a single CRC patient by mass spectrometry. Despite surgical resection of the primary CRC and adjuvant chemotherapy plus cetuximab treatment, the patient developed three metachronous CRLM which occurred consecutively after 9, 21 and 31 months. We identified a set of 1132 proteins expressed in the three metachronous CRLM, of which 481 were differentially regulated, including 81 proteins that were associated with the extracellular matrix (ECM). 56 ECM associated proteins were identified as upregulated in the third metastasis, 26 (46%) of which were previously described as negative prognostic markers in CRC, including tenascin C, nidogen 1, fibulin 1 and vitronectin. These data may reflect an ascending trend of malignancy from the first to the third metachronous colorectal cancer liver metastasis. Additionally, the results indicate different ECM phenotypes for recurrent metachronous metastasis, associated with different grades of malignancy and highlights the importance of individual analysis of molecular features in different, consecutive metastatic events in a single patient.

**Electronic supplementary material:**

The online version of this article (10.1007/s10585-020-10058-8) contains supplementary material, which is available to authorized users.

## Novelty and impact

In this study, we have identified for the first time differentially regulated proteins in three metachronous liver metastases from a single patient with colorectal cancer by mass spectrometric analysis. Regulated proteins indicate different ECM phenotypes for the three different metastases, whereas the third CRLM was associated with a significant upregulation of established negative prognostic biomarkers for CRC. The proteins identified in the recurrent metastases, including nidogen 1, tenascin C and vitronectin, may be useful as biomarkers for different CRLM phenotypes with different prognostic significance for individual metastatic recurrences in patients suffering from colorectal cancer.

## Introduction

More than 1.8 million cases of colorectal cancer (CRC) were estimated in the year 2018, making CRC the third frequent cancer burden worldwide. In addition, CRC patients suffer from the second highest mortality among all cancer entities [1] and colorectal cancer liver metastases (CRLM) can be observed in nearly half of CRC patients [2]. Due to the anatomically determined drain through the portal vein, the liver is the most common side for distant metastases of CRC [3]. In the clinical setting, the presence of liver metastases is associated with poor median survival rates ranging from 6 months without therapy to just 19 months under fluoropyrimidine and oxaliplatin (FOLFOX) or fluoropyrimidine and irinotecan (FOLFIRI) chemotherapy regimen [4]. Currently, the only curative treatment for CRLM remains R0 hepatic resection. Unfortunately, due to anatomically restrictions and extent of CRLM, hepatectomy in a curative intent is not always applicable. Especially in patients with recurrent liver metastases only one fifth match the technical requirements for repeated metastasectomy [4]. Apart from synchronous CRLM which occurs in 14% to 18% [5] of all CRC patients at primary diagnosis, there are also metachronous CRLM that can be observed after surgical treatment of the primary colorectal cancer [5]. Metachronous liver metastases are present in a lower number of patients ranging from 8 to 13% [5] after excision of the primary tumor. However, metachronous CRLM show a high frequency to recur in more than three quarters of all patients with prior CRLM surgery within a median interval of less than 14 months [4].

Given the lack of primary tumor burden, the cause of metachronous metastasis is still unclear. Nevertheless, previous studies could identify prognostic factors and molecular patterns that are solely associated with liver metastasis in colorectal cancer.

The extracellular matrix (ECM) consists of several protein groups containing different collagens, proteoglycans and glycoproteins. Distinct functions of the ECM include cell–cell communication, adhesion and regulation of proliferation processes that are known to be dysregulated in cancer progression [6]. In cancer development, the ECM undergoes dynamic remodeling inside its supramolecular aggregates required for cell migration, invasion and the progression of the cancer-specific microenvironment [7]. Altered expression of ECM proteins, e.g. the proteoglycan tenascin C (TNC), could be correlated to cancer among a wide variety of tumor entities, including colorectal carcinoma, underlining the importance of the extracellular matrix signatures in cancer [8].

Moreover, ECM remodeling, including the epithelial to mesenchymal transition are prerequisite for metastatic events and therefore the detachment of cells from the primary tumor. Consequently, ECM remodeling plays a crucial role in the formation of tumor and organ specific premetastatic niches and the adjustment of disseminated tumor cells to distant organs [9]. A recent study by Kim et al*.* identified 58 differentially regulated proteins in CRC primary tumors and their respective solitary synchronous CRLM of which many were associated with the ECM [10], suggesting a crucial role of ECM proteins in colorectal cancer liver metastasis.

In the present study, we analyzed the protein composition of three recurrent metachronous liver metastases and the healthy liver tissue of one patient with colorectal cancer using mass spectrometry. The metastases were treated 9, 21 and 31 months after first presentation. While the first two metachronous CRLM were found in the right liver lobe (liver segment V and IV/VIII, respectively), the third one was located in the left liver lobe (liver segment II/IVa). Chemotherapy was applied two times, once after identification of the primary tumor in the FOLFOX regimen combined with anti-epidermal growth factor receptor antibody cetuximab. FOLFOX chemotherapy scheme was repeated after the first occurrence of liver metastasis.

## Materials and methods

### Patient’s characteristics

The male patient was diagnosed with colorectal cancer in September 2010 at the age of 59 years. The patient was treated with neoadjuvant radio chemotherapy followed by abdominoperineal resection of the rectum (TNM: ypT2 N0 (0/14) G2 L0 V0 M0 R0) and four cycles of adjuvant 5-FU in December 2010. Nine months later, in June 2011, the patient presented with the first metachronous liver metastases (segment V) and was treated with FOLFOX regimen and cetuximab preoperatively for 3 months, followed by right hemihepatectomy (sample M1) in September 2011 and another 3 months of FOLFOX regime after surgery. In May 2012, the patient relapsed with recurrent liver metastases in liver segment IV / VIII. The patient received six cycles of FOLFIRI in combination with bevacizumab for 4 months, with subsequent resection (sample M2) of a liver metastasis from segment IV in September 2012. 34 months after the first presentation with CRC, the patient developed a third recurrent metachronous liver metastasis (sample M3) in June 2013, which was treated with subsequent atypical resection of segment II/IVa without admission of further chemotherapy in July 2013. The patient had a further tumor relapse in 2014 in liver segment III, which was not resected, and died in the same year due to pulmonary embolism.

Informed consent was obtained from the patient. All experiments involving material of human origin were performed in accordance with relevant guidelines and regulations given by the local authorities and regional science ethics committee.

### Tissue sectioning and preparation

20 consecutive frozen tissue sections were prepared with a microtome in 6 µm thickness and transferred onto glass slides. Adjacent sections before and after were H&E-stained for histologic examination. The tissue sections were rinsed twice in 100% ethanol, and once each in 95%, 70% and 30% ethanol.

### Tryptic digestion

Tissue sections were transferred into new reaction vials containing 200 µL of 10 mM dithiothreitol dissolved in 100 mM ammonium bicarbonate solution for reduction of cysteine residues and incubated for 10 min at 56 °C. Cysteines were then alkylated with 300 mM iodoacetamide and incubated in the dark at room temperature for 20 min. Finally, proteins were digested overnight at 37 °C using 200 µL sequencing-grade-modified trypsin at a final concentration 0.1 µg/µL (Promega Corporation, Madison, USA) dissolved in 40 mM ammonium bicarbonate. The samples were centrifuged at 12,000 rpm for 10 min and the supernatants were evaporated to ensure complete dryness.

### Mass spectrometric analysis

Lyophilized peptides were resuspended in 20 µL of 0.1% formic acid and centrifuged for 5 min at 15,000 rpm at 4 °C. The tryptic peptides were analyzed on a reversed-phase nano-UPLC (Dionex UltiMate 3000 RSLCnano, Thermo Fisher Scientific, Bremen, Germany) coupled via electrospray ionization to an orbitrap mass spectrometer (Orbitrap Fusion, Thermo Fisher Scientific). Samples were loaded on a trapping column (Acclaim PepMap μ-precolumn, C18, 300 μm × 5 mm, 5 μm, 100 Ǻ, Thermo Scientific) at 98% solvent A (0.1% formic acid in HPLC water) and 2% solvent B (0.1% formic acid in acetonitrile), and separated on the separation column (Acclaim PepMap 100, C18, 75 μm × 250 mm, 2 μm, 100 Ǻ, Thermo Fisher Scientific) with an elution gradient (2–30% B in 90 min, 30–70% B in 10 min, 70–90% B in 2 min) at a flow rate of 200 nL/min. The mass spectrometer was operating in data-dependent acquisition top speed mode (cycle time 1 s), detecting positive ions in an *m*/*z* scan range of 400–1500 at resolution of 120,000 at 200 m/z (AGC target 4e5, maximum injection time 50 ms). For fragmentation, signals with charge states 2 to 6 were isolated with a 1.5 m/z window, fragmented at a normalized HCD collision energy of 35 and detected in the linear ion trap in rapid mode, covering a mass range of 350–1400 (AGC target 1e4, maximum injection time 50 ms).

### Peptide identification and protein quantification

Mass spectrometric data was analyzed with MaxQuant 1.6.3.4 [11]. The peptide fragment spectra were identified with the Andromeda search engine against the human SwissProt database (www.uniprot.org, downloaded April 24, 2018; 20,316 entries) and the internal contaminant database. The precursor and fragment mass tolerances were set to 10 ppm and 0.2 Da, respectively. Two missed cleavages were allowed. Carbamidomethylation on cysteine residues was set as a static modification, and oxidation of methionine residues and acetylation of the N-terminus as variable modifications. Peptides and proteins were identified with an FDR of 1%. Proteins were quantified with the MaxLFQ algorithm [12] considering only the unique peptides of each protein. For the following analyses, proteins identified with at least two unique peptides in total were included.

### Proteomics data analysis

Analysis of proteomics data was carried out with Perseus 1.6.2.3 [13]. Label-free quantification (LFQ) intensities transformed to common logarithm (base 10). Unsupervised, pearson correlation-based hierarchical clustering with average linkage was used to visualize similarities and differences between technical replicates, in different metachronous CRLM and adjacent liver tissue. Two-sided t-testing, with permutation-based FDR correction (q-value < 0.05) was performed for pair-wise comparisons of among the metastases. Proteins that showed difference in abundance of at least twofold in at least one pair-wise comparison, as well as proteins that were exclusively identified in one sample were considered as regulated.

Enrichment analysis of regulated proteins was performed with DAVID 6.8 [14, 15] using the list of all identified proteins as the background. The functional annotation chart and the functional annotation clustering tool were used to find enriched terms and pathways.

The tissue distribution of regulated proteins was annotated using the normalized-consensus-transcript-expression-dataset provided by the human protein atlas [16]. The ECM association of regulated proteins as well as their prognostic value in CRC were investigated by a National Center for Biotechnology Information (NCBI) library-based search using the ‘OmixLitMiner’ tool for literature retrieval [17] (‘extracellular matrix’ in title or abstract). Afterwards, ECM association was annotated manually. ECM proteins were visualized based on a STRING protein–protein interaction map. Experiments, databases and co-expression were used as active interaction sources at a minimum required interaction score of 0.9. The mass spectrometry proteomics data have been deposited to the ProteomeXchange Consortium via the PRIDE (https://www.ebi.ac.uk/pride/) partner repository with the dataset identifier PXD020109.

## Results and discussion

**Fig. 1:**
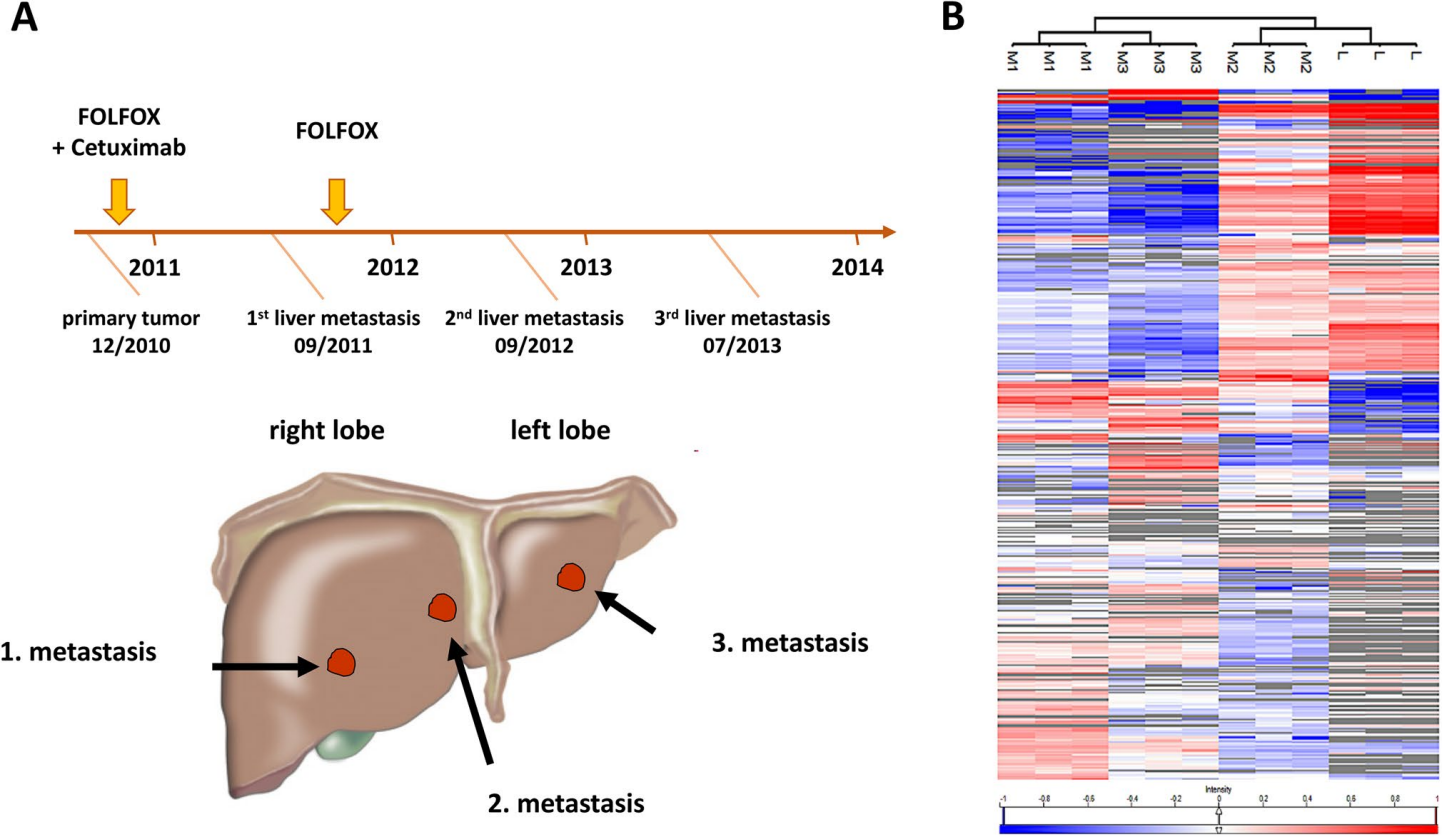
Clinical history of the patient and a heat map of unsupervised hierarchical clustering based on all 1173 proteins identified. **A** Clinical appearance, treatment history and localization of three metachronous liver metastases from a single CRC patient. In total, the patient developed three metachronous liver metastases, which were treated 9, 21 and 31 months after presentation. The patient died in 2014 due to pulmonary embolism. **B** Pearson correlation-based, unsupervised hierarchical clustering based on all 1173 proteins identified in the three metastases and the healthy liver samples adjacent to the first metastasis, each with three technical replicates. Average linkage was used as distance metric. Protein abundances were transformed to common logarithm (log10) and the median values normalized across the columns. Mean value normalization was performed across rows for better visualization. Grey cells indicate that a protein could not be identified in the respective sample. M1, metastasis 1; M2, metastasis 2; M3, metastasis 3; L, healthy liver tissue

Three metachronous liver metastases from a single patient with colorectal cancer (Fig. 1A) and a sample from adjacent healthy tissue of the first metastasis (M1) were subjected to label-free quantitative proteomics. A total of 1173 proteins were identified in the four samples in triplicates, of which 1132 were present in at least one of the metastases (Suppl. Table 1, Suppl. Fig. 1). The three metachronous CRLM express a unique set of 39, 93 and 19 proteins, respectively (Suppl. Fig. 1, Suppl. Table 2).

In the healthy tissue adjacent to the first metastasis, 196 proteins were significantly upregulated when compared to the metastases, of which 125 proteins (63.8%) were annotated with the ‘liver’ keyword in the ‘UniProt tissue’ database. Conversely, the 'epithelium' annotation was highly significantly enriched among the proteins that were upregulated in the metastasis (149 out of 426 proteins, 35%) (Suppl. Table 3), underlining the different origins of both tissue types i.e. liver parenchyma and colon epithelium.

**Fig. 2:**
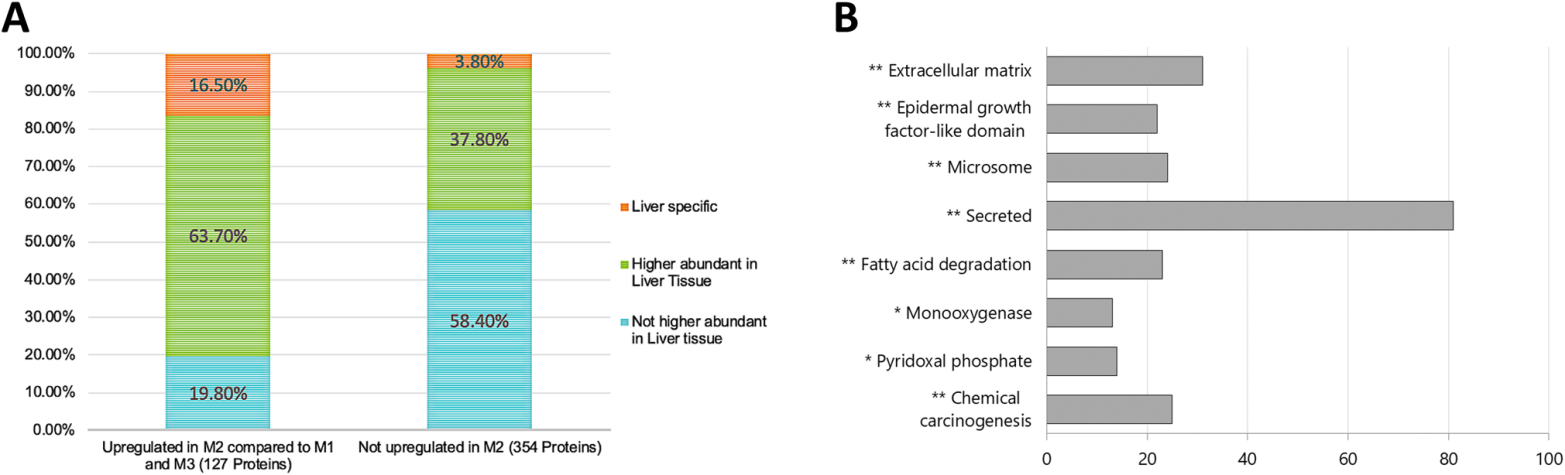
Annotation of 481 proteins, identified as differentially regulated in pair-wise comparisons among the metastases*.* **A** Known gene expression levels of differentially regulated proteins in liver tissue, compared to 54 tissue types (including colon tissue) and 6 blood cell types, were annotated according to the normalized consensus transcript expression dataset provided by the Human Protein Atlas. Due to the clustering of M2 with liver tissue, regulated proteins were divided into two bars (proteins upregulated in M2 compared to M1 and M3 and proteins not upregulated in M2, respectively). M1, metastasis 1; M2, metastasis 2; M3, metastasis 3. **B** Bar chart of relevant enriched keywords in regulated proteins identified by the DAVID enrichment analysis. The bars indicate the number of proteins associated with the corresponding keyword. Benjamini-Hochberg-corrected p values < 0.05 were considered as statistically significant (*p < 0.05; **p < 0.01)

In the unsupervised hierarchical clustering, the second metastasis (M2) showed a higher similarity to liver tissue than to the first and the third (M3) metachronous CRLM (Fig. 1B). Furthermore, 106 out of 127 proteins (83%), upregulated in M2 compared to M1 and M3, had the highest tissue-specific expression in liver tissue, according to the human protein atlas (Fig. 2A). The difference can be partly explained by the slightly lower tumor content of the M2 (40% vs 50%). Nevertheless, it is notable that the higher similarity of M2 to liver tissue seemingly does not significantly alter the ECM profile as M2 shows a higher ECM similarity to M1 and M3. Additionally, since the liver-specific proteins were upregulated more than twofold, adaptation of M2 to liver tissue is likely. Adaptation of cancer cells to their respective metastatic sites has been reported among a wide variety of tumor entities, including breast cancer [18, 19]. Similarly, the ability of CRLM to adopt features of the liver-specific microenvironment, such as extensive upregulation of the fructose metabolism due to metabolic reprogramming, has been described previously [20].

In pair-wise comparisons between the three metastases, a total of 481 proteins (41%) were found to be differentially regulated (Suppl. Table 4). Enrichment analysis with the DAVID functional annotation clustering tool revealed significant enrichments of various notable keywords (Fig. 2B) including extracellular matrix (31 out of 481 proteins), epidermal growth factor-like domain (22 proteins), and microsome (24 proteins). Further enriched keywords were secreted (81 proteins), monooxygenase (13 proteins), fatty acid degradation (23 proteins), pyridoxal phosphate (14 proteins), and chemical carcinogenesis (25 proteins) (Fig. 2B, Suppl. Table 5).

**Fig. 3:**
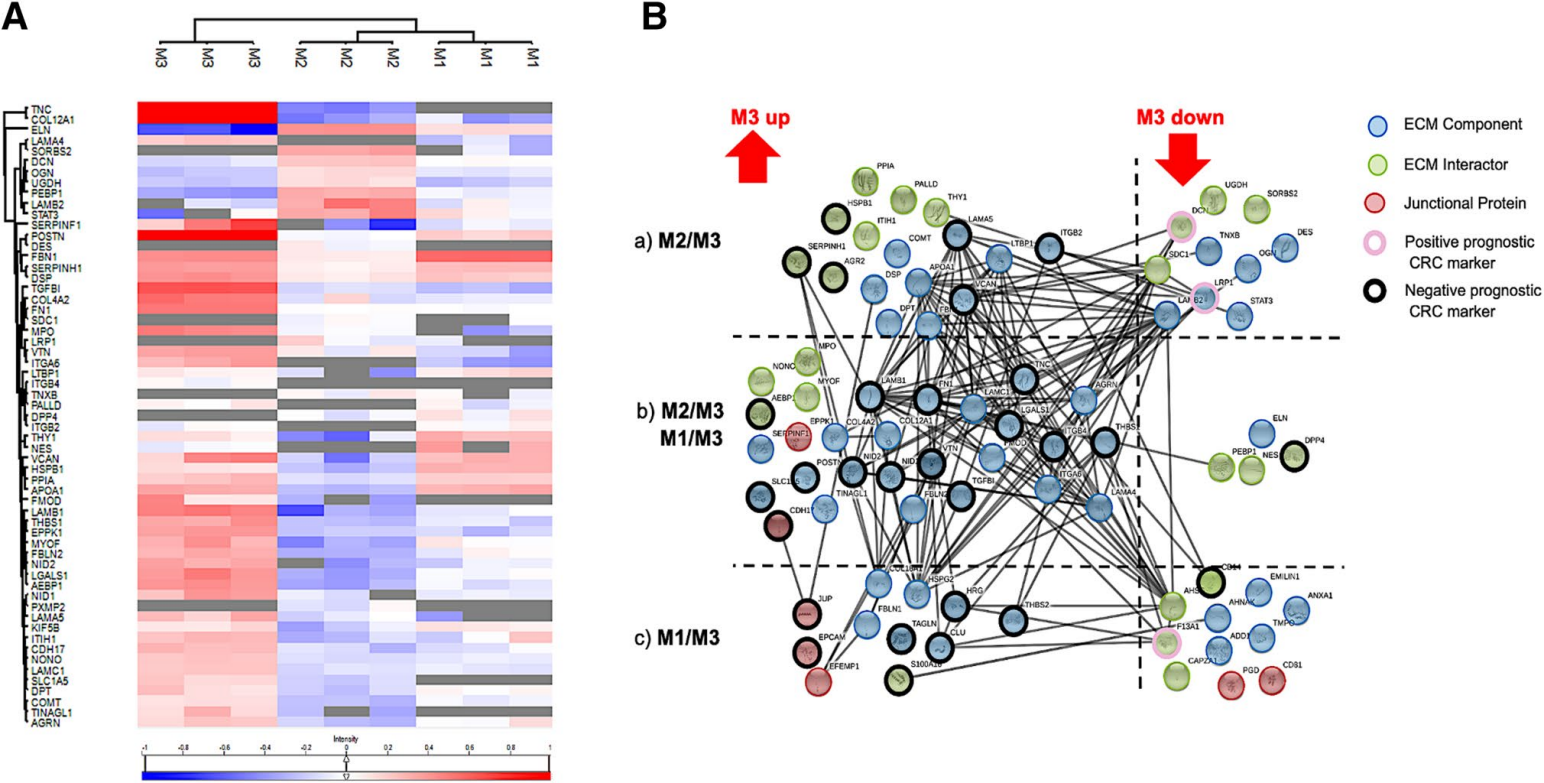
Detailed analysis of the differently regulated ECM-associated proteins in three metachronous CRLM of a single patient. 81 ECM-associated proteins were identified in the subset of regulated proteins (481). **A** Heat map of pearson correlation-based, hierarchical clustering of differentially regulated ECM associated proteins. Average linkage was chosen as distance metric. Protein abundances were transformed to common logarithm (log10) and the median values normalized across the columns. Mean value normalization was performed across rows for better visualization. Grey cells indicate that a protein could not be identified in the respective sample. **B** Modified, confidence-based STRING protein–protein interaction map. Experimental evidence, association in curated databases and co-occurrence were used as interaction sources. The minimal required interaction score was set to 0.9 (highest confidence). Proteins were grouped into three categories (ECM component, blue; ECM interactor, green; junctional protein, red) and then sorted according to the metastases between which they were identified as regulated: (a) Exclusively regulated between M2/M3; (b) regulated between M1/M3 and M2/M3; (c) exclusively regulated proteins between M1/M3. For known prognostic markers in CRC, the prognostic significance was annotated (positive prognostic marker, red circle; negative prognostic marker, black circle; unknown significance, no circle). M1, metastasis 1; M2, metastasis 2; M3, metastasis 3

Remodeling of the extracellular matrix has been described as a requirement for metastatic growth in the past. Furthermore, the regulation of ECM components significantly impacts the clinical outcome in different tumor entities, including CRC [21], in which differential ECM signatures among metachronous metastasis from one patient are of particular interest. An automated as well as manual knowledgebase search was performed to obtain a comprehensive coverage of known associations of regulated proteins with the ECM. A heatmap of all 81 regulated proteins, associated with the keyword ‘extracellular matrix’ is shown in Fig. 3A and Supplementary Table 6.

A total number of 33 regulated ECM proteins, were found to be described as prognostic markers for CRC in literature (Suppl. Table 8). Strikingly, 56 proteins (70%) were significantly higher abundant in the third metastasis, including 28 proteins whose upregulation has been previously linked to impaired survival rates in cancer (Suppl. Table 8). The identified proteins include tenascin C (TNC), nidogen1 (NID1) and vitronectin (VTN) (Fig. 3B, Suppl. Table 7).

The ECM glycoprotein TNC, has been reported to be a major driver of invasive colorectal carcinoma promoting liver metastasis, underlining the importance of tumor-stroma interactions and suggesting TNC as a novel biomarker in primary CRC [22]. NID1 has been found to be solely upregulated in the third metachronous CRLM the patient developed. In the past, NID1 was found to be capable of inducing epithelial-mesenchymal transition in epithelial-like CRC cells. Additionally, evidence of increased NID1 expression could be observed in primary CRC and correlated with impaired patient survival and increased tumor proliferation [23]. The ECM protein VTN [24], has been previously described as a potent migratory enhancing factor for cancer cells. An inhibition of its pro-migratory capacity is achieved upon formation of an inhibitory complex with fibrinogen [25].

In addition, the upregulation of single ECM associated proteins, different ECM associated signatures and metastatic phenotypes across different patients have been described previously in CRLM [26]. In 2001, Lunevicious et al. [26] reported histopathological differences in CRLM, namely metastasis with fibrotic encapsulation and without fibrotic capsule. Lack of fibrotic encapsulation was associated with poor prognosis and reduced survival [26]. Strikingly, a high amount of proteins associated with non-fibrotic CRLM [27], including TNC, fibronectin, different collagens, integrins and laminins, were found to be highly upregulated in M3 (Fig. 3B).

In addition to that, the TGF-β inhibitor (TGFI) was found to be upregulated in our third CRLM. TGFI inhibits the transforming growth factor β (TGF-β) signaling pathway, which plays a crucial role in fibrotic events as reported previously [28]. In line with the poor prognosis and lower overall survival rates reported for non-encapsulated CRLM [29], the upregulation of TGFI may be a hint towards a higher malignancy of the third and also last CRLM of the patient before he succumbed to the disease. Furthermore, in M3 in contrast to M1 and M2, we also observed a downregulation of laminin subunit beta-2 (LAMB2), decorin (DCN) and coagulation factor XIII A chain (F13A1), which are said to be associated with good prognosis in CRC (Fig. 3B, Suppl. Table 8). Upregulation of negative prognostic markers with consecutively downregulation of positive prognostic markers in line with chronologically development of the metastasis, may implicate an increased malignancy of the third metachronous CRLM compared to first two CRLM the patient developed.

Our data thus suggests, to our best knowledge, for the first time, different ECM phenotypes of different metachronous CRLM that have been developed in a single patient. As already reported the different metastatic ECM phenotypes are obviously associated with different prognosis and survival probabilities.

Data on recurrent metachronous colorectal carcinoma liver metastases are scarce but of high clinical importance as they are crucial for prognosis. In our study, we identified numerous differentially regulated proteins in three recurrent metachronous CRLM of the same patient. These finding are in line with the current literature indicating that differentially regulated ECM proteins play a functional role in the metastatic cascade of colorectal carcinoma [30, 31]. The approach can be interpreted as a proof of principle that may be extensively studied in a larger cohort to acquire more knowledge on different ECM signatures of metachronous metastases in colorectal cancer. Especially data on the functional role and clinical impact of ECM signatures is necessary to improve treatment of patients suffering from recurrent metachronous CRLM. Additionally, comprehensive molecular analysis of metastases from different points in time during disease progression should be performed. Identification of ECM signatures may be useful as biomarkers to predict metachronous CRLM and different CRLM phenotypes that can impact prognosis and improve the selection of optimal therapeutic measures individually for each metastatic event. Identification of predictive and prognostic biomarkers for CRLM by mass spectrometric analysis may be a valuable approach for patients suffering from colorectal cancer.

## Electronic supplementary material

Below is the link to the electronic supplementary material.

Supplemental Figure 1 Venn diagram showing the number of proteins found in all three technical replicates of the three metastases. 1,132 proteins were found in the three metastases in total. A unique set of 39, 93 and 19 proteins were detected in the three metachronous CRLM, respectively. (PDF 57 kb)

**Supplemental Table 1** List of all identified proteins (1,173) by mass spectrometry with at least two unique peptides in total. **Supplemental Table 2** List of proteins which were detected exclusively in one of the three recurrent metastases. In our analysis 39, 93, and 19 proteins were detected exclusively in one of the three colorectal cancer liver metastases M1 to M3, respectively. **Supplemental Table 3** List of enriched 'UniProt tissue' keywords among the proteins that were up- or downregulated between the first metastasis (M1) and the surrounding healthy liver tissue adjacent to M1 as identified by the DAVID enrichment analysis with the functional annotation chart tool. **Supplemental Table 4** List of all 481 differentially expressed proteins in the three colorectal cancer liver metastases. **Supplemental Table 5** List of relevant enriched keywords in the 481 proteins which were differentially expressed among the three recurrent metastases identified by the DAVID enrichment analysis with the Functional Annotation Clustering tool. **Supplemental Table 6** List of 81 differentially regulated proteins, classified as ECM associated proteins according to manual literature research. ‘OmixLitMiner’ was used to retrieve all PubMed listed publications that connect a protein to the keyword ‘extracellular matrix’ in title or abstract. The source publications used for the ECM association are shown. **Supplemental Table 7** List of differentially regulated ECM proteins, previously described as prognostic marker in CRC (32 proteins), according to manual literature research. OmixLitMiner was used to retrieve all PubMed listed publications that connect a protein to the keyword ‘colorectal carcinoma’ in title or abstract. The source publications used for the colorectal cancer association are shown. (XLSX 1193 kb)

Supplementary material 3 (DOCX 13 kb)

## Data Availability

All data generated or analyzed during this study are included in this published article. The mass spectrometry proteomics data have been deposited to the ProteomeXchange Consortium via the PRIDE (https://www.ebi.ac.uk/pride/) partner repository with the dataset identifier PXD020109.

## Abbreviations

5-FU: 5-Fluorouracil
CRC: Colorectal cancer
CRLM: Colorectal cancer liver metastases
DCN: Decorin
ECM: Extracellular matrix
F13A1: Coagulation factor XIII A chain
H&E: Haematoxylin and eosin
M1: First metastasis
FOLFIRI: Fluoropyrimidine and irinotecan
FOLFOX: Fluoropyrimidine and oxaliplatin
LAMB2: Laminin subunit beta-2
NID1: Nidogen 1
M2: Second metastasis
TNC: Tenascin C
M3: Third metastasis
TGFI: Transforming growth factor beta inhibitor
TGF-β: Transforming growth factor beta
VTN: Vitronectin
