# Supplementary figures and images for: Differential regulation of extracellular matrix proteins in three recurrent liver metastases of a single patient with colorectal cancer
Source: Clin Exp Metastasis. 2020 Oct 24;37(6):649–56. doi: 10.1007/s10585-020-10058-8 (PMC7666585; doi:10.1007/s10585-020-10058-8)

Suppl. Figure 1

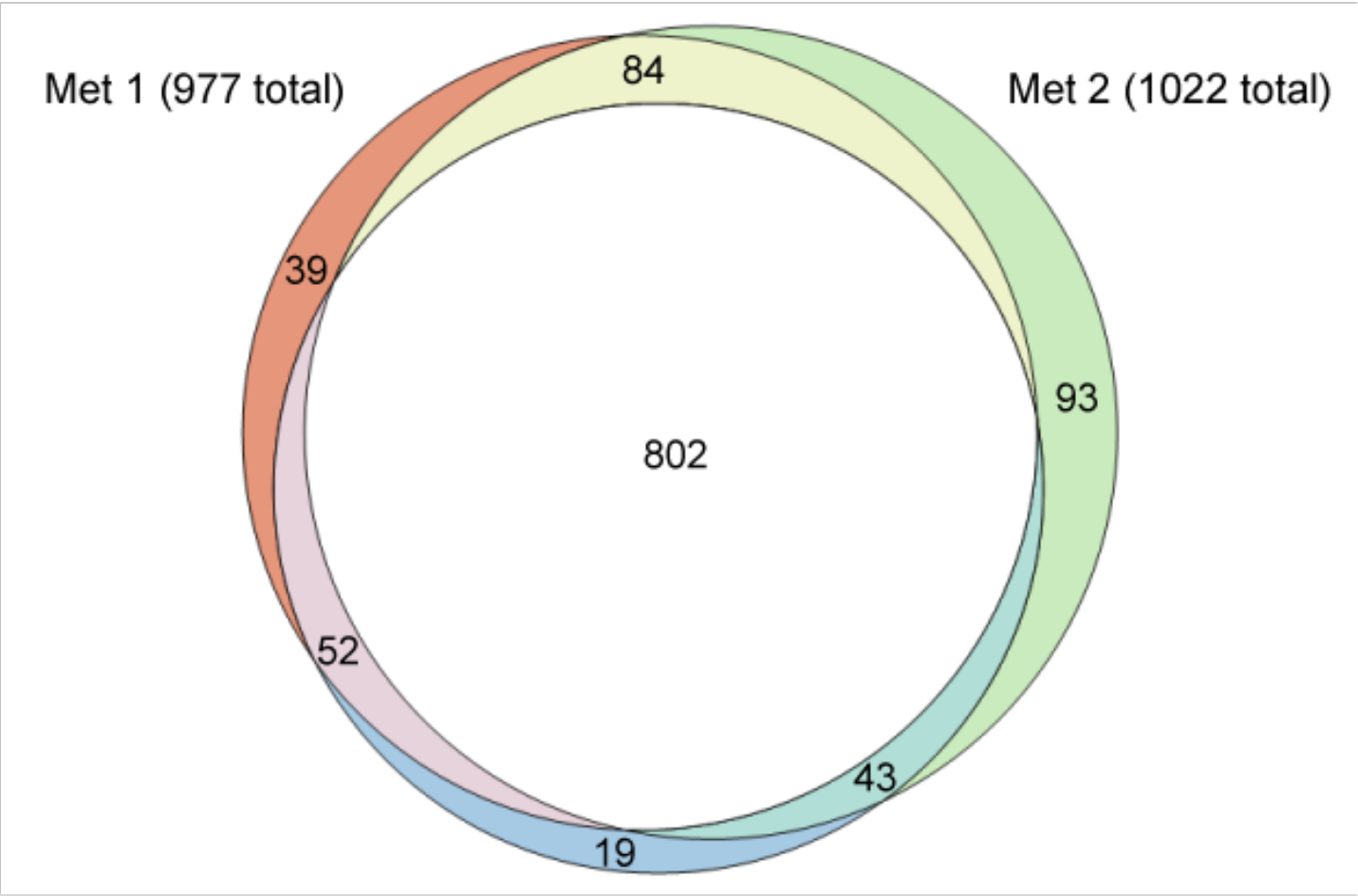

Met 3 (916 total)

Supplement: Supplementary file 1 — Venn diagram showing the number of proteins found in all three technical replicates of the three metastases. 1,132 proteins were found in the three metastases in total. A unique set of 39, 93 and 19 proteins were detected in the three metachronous CRLM, respectively. (PDF 57 kb) [file 10585_2020_10058_MOESM1_ESM.pdf]
