# Supplementary material for: Differential regulation of extracellular matrix proteins in three recurrent liver metastases of a single patient with colorectal cancer
Source: Clin Exp Metastasis. 2020 Oct 24;37(6):649–56. doi: 10.1007/s10585-020-10058-8 (PMC7666585; doi:10.1007/s10585-020-10058-8)
Supplement: Supplementary file 3 — Supplementary material 3 (DOCX 13 kb) [file 10585_2020_10058_MOESM3_ESM.docx]

**Supplemental Material**

**Supplemental Figure 1:** Venn diagram showing the number of proteins found in all three technical replicates of the three metastases. 1,132 proteins were found in the three metastases in total. A unique set of 39, 93 and 19 proteins were detected in the three metachronous CRLM, respectively.

**Supplemental Table 1:** List of all identified proteins (1,173) by mass spectrometry with at least two unique peptides in total.

**Supplemental Table 2:** List of proteins which were detected exclusively in one of the three recurrent metastases. In our analysis 39, 93, and 19 proteins were detected exclusively in one of the three colorectal cancer liver metastases M1 to M3, respectively.

**Supplemental Table 3:** List of enriched 'UniProt tissue' keywords among the proteins that were up- or downregulated between the first metastasis (M1) and the surrounding healthy liver tissue adjacent to M1 as identified by the DAVID enrichment analysis with the functional annotation chart tool.

**Supplemental Table 4**: List of all 481 differentially expressed proteins in the three colorectal cancer liver metastases.

**Supplemental Table 5:** List of relevant enriched keywords in the 481 proteins which were differentially expressed among the three recurrent metastases identified by the DAVID enrichment analysis with the Functional Annotation Clustering tool.

**Supplemental Table 6:** List of 81 differentially regulated proteins, classified as ECM associated proteins according to manual literature research. ‘OmixLitMiner’ was used to retrieve all PubMed listed publications that connect a protein to the keyword ‘extracellular matrix’ in title or abstract. The source publications used for the ECM association are shown.

**Supplemental Table 7:** List of differentially regulated ECM proteins, previously described as prognostic marker in CRC (32 proteins), according to manual literature research. OmixLitMiner was used to retrieve all PubMed listed publications that connect a protein to the keyword ‘colorectal carcinoma’ in title or abstract. The source publications used for the colorectal cancer association are shown.
